# Taste sensitivity throughout age and the relationship with the sleep quality

**DOI:** 10.5935/1984-0063.20190127

**Published:** 2020

**Authors:** Maria Eduarda Martelli, Natália Jacob, Milca Abda Morais, Diogo T da-Cunha, Ligiana P Corona, Caroline Dário Capitani, Andrea Maculano Esteves

**Affiliations:** Universidade Estadual de Campinas, Faculdade de Ciências Aplicadas - Limeira - São Paulo - Brazil

**Keywords:** Aging, Taste Perception, Sleep

## Abstract

**Objective:**

The aim of the present study was to evaluate taste sensitivity and sleep pattern throughout age.

**Methods:**

Thirty-five male adults aged (25.05±0.71 years), and twenty- four older adults (68.92 ± 6.43 years) were selected and submitted to sleep evaluation (Pittsburgh Sleep Quality Index and Epworth Sleepiness Scale), as well as taste sensitivity. Taste sensitivity was evaluated using three dilutions and the different concentrations were presented for the four basic flavors (salty, sweet, bitter and sour). These samples were encoded with three digits and randomly presented to the participants in 50 mL plastic cups.

**Results:**

In both groups, sleep quality was poor (PSQI >5). Older adults presented a negative effect to identify sweet and salty taste and, in this population, sleep time was associated with sweet taste perception.

**Discussion:**

We may suggest that aging may cause changes in taste sensitivity, as well as total sleep time was observed to be a significant predictor of sweet taste. Sleep may play an important role in taste sensitivity, although the mechanisms are still unknown. Thus, the results of this research may contribute to the emergence of new studies that seek to better understand this relationship of sleep quality as taste sensitivity.

## INTRODUCTION

Aging changes occur in all of the body’s cells, tissues and organs, and these changes affect the functioning of all body systems. Thus, physiological aging is accompanied by alterations in the quality, quantity and architecture of the sleep, being able to lead to diverse diseases^1^, such as diabetes^2^, and increase of the risk of depression and anxiety^3^, for example. Consequently, older people would use more health care services, generating demands that many health systems are not prepared to meet, especially in developing countries, deepening social and economic problems^4^.

Some of the changes in sleep architecture seen in aging are: increased time spent in bed without sleeping, difficulties to restart sleep, less night sleeping time, increased sleep latency, reduction of sleep efficiency and slow-wave, early awakening and increased daytime sleepiness and fatigue^5,6^. Besides to changes in sleep pattern, it is known that aging can also cause anatomical and physiological modifications, related to variations in taste sensitivity, such as atrophy and reduction of taste^7^, decreased sensory perception, changes in the functioning of the digestive tract, in masticatory efficiency, salivary flow and integrity of the oral mucosa^8^.

Gustatory sensitivity occurs through receptor cells (taste buds), which are found in greater number in the epithelium of the tongue, and to a lesser extent in other regions, such as palate, cheeks and esophagus. The taste buds confer specificity to gustatory stimuli, which allows the recognition of basic tastes: salty, sweet, bitter, sour and umami^9^.

In aging, loss of taste is more pronounced for bitter and salty tastes^10^. In addition, the reduction in saliva volume and salivary amylase activity, hinders the initial digestion of carbohydrates; this may contribute to less sweet taste perception, and generate glucose changes^11^. Some genetical polymorphisms also give rise to large inter-individual differences in taste perception especially in elderly women^12^.

In addition, some studies suggest that changes in sleep pattern may modify chemosensory function^13,14^, as well as a possible correlation between sleep restriction and feelings of hunger, food choice and caloric intake^14-16^. However, how sleep architecture can contribute to differences in taste preference is still unknown^17^. Despite the uncertainty of the exact mechanisms, sleep is known to play an important role in biological homeostasis, particularly in the endocrine system^17^, which influences taste sensitivity, especially in the older adults. Thus, considering the limited number of studies and the remaining gaps, this study is of great importance to open new ways for a better understanding between the relationship of changes in sleep pattern and taste sensitivity in the elderly population.

Thus, considering that the older adults population may present changes in sleep quality^1^ as well as taste sensitivity^7^, it has been demonstrated relationship between these two factors^18^; and in addition, such factors may be related to reduced quality of life and increased chance of developing some diseases^1^, the aim of the present study was to verify if taste sensitivity can be altered throughout the age and if the quality of the sleep contributes to such alteration.

## METHODS

### Sample selection

The selected sample consisted of adults (N=35) and older adults (N=24) population. The experimental design of this study was approved by the Ethics in Research Committee (#1424822 and #1840584) and was conducted by the Declaration of Helsinki. All participants gave written informed consent before taking part.

The adult population was selected among the employees, teachers and students of a public college. Interested volunteers filled out a screening and selection form that contained personal information, use of supplements and medications, and health history. The exclusion criteria for adults were use of any type of medication, age over 60 years, presence of chronic disease and tobacco. The woman was considered an exclusion factor due to hormonal oscillations. The recruitment and selection of the older adults was carried out at village for low-income older adult’s individuals, constituted as a senior living community of dwelling and areas of social coexistence. The inclusion criteria were: age between 65 and 75 years, reside in village. Initially, 26 residents were recruited; however, 2 of them refused to participate in the research, and then 24 were selected. For older adults, the only exclusion criteria were tobacco, while that the older adults who used medication or who had chronic disease treated were not excluded from the study.

### Experimental design

A cross-sectional study was carried-out. The data collection occurred in two moments, in different days. At the first moment, besides the presentation and signing of the Informed Consent Term (ICT), anamnesis was performed, with questionnaires for sleep evaluation and anthropometric measurements. At the second moment (after one week), the taste sensitivity test was performed.

## EXPERIMENTAL PROCEDURE

### Physical assessments

The measures of stature and body mass were obtained by means of stadiometer (SECA, model 213) and calibrated digital scale (BK - 50F - Balmak), respectively. The Body Mass Index (BMI) was calculated by dividing the body weight (kg) by the square of height (m).

### Sleep pattern

For evaluation of sleep the following questionnaires were used:

Sleep quality index was obtained through Pittsburgh Sleep Quality Index (PSQI), of Buysse et al.^19^, already validated and translated into the Portuguese language (PSQI-BR) by Bertolazi et al. This questionnaire consists of 21 items that evaluate the quality of sleep and its disorders, through the previous month registry, by means of the following components: sleep latency, sleep duration, sleep efficiency, sleep disturbances, use of medications and dysfunctions during the day. The classification criterion was based on the total score obtained by sorting the sleep of the participants as good (≤ 5 points) or poor quality of sleep (> 5).

### Evaluation of sensitivity taste

Participants were advised to maintain a healthy diet and not to use alcohol the day before the test. The samples used were prepared one day before the test, in the Laboratory of Technic and Dietary at the School of Applied Sciences, UNICAMP, and were kept under refrigeration at a temperature range between 0 to 10 degrees Celsius. These samples were encoded with three digits and randomly presented to the participants in 50mL plastic cups^21^.

The sample concentration was determined according to the Dutcosky^21^ using the following dilutions:

Salty taste (S): sodium chloride was used in concentrations of 0.02%, 0.12% and 0.22%.

Sweet taste (SW): refined sugar cane was used in the following concentrations: 0.4%, 0.6%, and 0.8%.

Bitter taste (B): caffeine (p.a) was used in growing concentrations of 0.01%, 0.02% and 0.03%.

Sour taste (SO): citric acid was used in the following concentrations: 0.03%, 0.04% and 0.05%.

All concentrations, for all tastes, were dissolved in distilled water. After the delivery of each concentration, one offered 50mL of distilled water and waited 20 seconds for the presentation of the next sample^21^.

### Data analysis

The statistical tests have been adopted in accordance with the distribution of the data (parametric and non-parametric). The Student’s t-test was used for parametric data analysis, for independent samples, and for non-parametric data we used Mann-Whitney’s U Test. To test variables adherence to the normal distribution Shapiro-Wilk test was used.

A Principal Component Analysis (PCA) was done to explore the components of taste sensitivity and reduce into fewer groups. It was done a PCA with Varimax Rotation and Kaiser Normalization. It was considered only factors with loadings above 0.40.

The confirmatory analysis was done later to evaluate the reliability of each component. It was considered adequate components with Cronbach’s alpha greater than 0.55.

Four multiple linear regression were done to determine which variables were associated with the different tastes (i.e. salty, sweet, bitter and sour). The independent variables in the model were those variables that presented a Pearson correlation coefficient greater than 0.30. The variables insertion in the models was done by Stepwise method with forward selection. The independent variables remained in the model if they were statistically significant (*p*<0.05). Homoscedasticity and model fit were evaluated by residual analysis. The data analysis was performed using SPSS version 15.0.

The level of significance was *p*≤0.05 and the data were expressed as mean ± standard deviation.

## RESULTS

The adult sample consisted of 35 males; the mean age was 25.05 years old with BMI of 25.1kg/m². The older adult’s sample was composed of 24 males, had 68.92 years old and mean BMI of 28.7 kg/m². The sleep of the adult’s and older adults are shown in Table 1. The most of adults (68.5%), as well as the older adults (54.16%), reported poor sleep quality and (PSQI>5). In addition, volunteers classified as having poor sleep quality had a statistically significant reduction in sleep efficiency compared to those with good sleep quality.

**Table 1:** Sleep characteristics of participants and comparison of good and poor sleep quality.

Variables Sleep Quality	Age group	General	Good Sleep Quality	Poor Sleep Quality	*p*
Pittsburgh Score	Adults	6.14 ± 2.51	3.36 ± 1.02	7.41 ±1 .86	<0.0001
	Older Adults	5.37 ± 3.12	2.83 ± 1.46	7.91 ± 1.83	<0.0001
Latency (min) *	Adults	17.45 ± 11.98	15.68 ± 7.91	18.27 ± 13.52	0.93
	Older Adults	21.75 ± 20.36	15.58 ± 16.22	27.91 ± 20.91	0.21
Efficiency (%)	Adults	94.33 ± 8.35	98.63 ± 7.82	92.37 ± 7.98	0.03
	Older Adults	94.17 ± 22.65	105.33 ± 16.25	83.01 ± 23.62	0.01

Data expressed as mean ± standard deviation; Student's t-test for independent samples;

* Mann-Whitney Test.

The factorial analysis resulted in four well-defined components for each taste. The sweet taste (0.8%) was removed because presented factor loading less than 0.40.

The components presented reasonable reliability with their respective Cronbach’s alpha: Component 1 (Sour component): 0.79; Component 2 (Bitter component): 0.69; Component 3 (Salty component): 0.74; and Component 4 (Sweet component): 0.57 (Table 2).

**Table 2:** Principal component analysis with different tastes and concentrations.

Variable	Component			
	1	2	3	4
Salty taste (0.02%)	-	-	.765	-
Salty taste (0.12%)	-	-	.854	-
Salty taste (0.22%)	-	-	.793	-
Sweet taste (0.4%)	-	-	-	.838
Sweet taste (0.6%)	-	-	-	.779
Sweet taste (0.8%)	-	-	-	-
Bitter taste (0.01%)	-	.682	-	-
Bitter taste (0.02%)	-	.898	-	-
Bitter taste (0.03%)	-	.727	-	-
Sour taste (0.03%)	.794	-	-	-
Sour taste (0.04%)	.873	-	-	-
Sour taste (0.05%)	.819	-	-	-

Table 3 presents the results of the regression models. The models for the sour and bitter taste were not significant. It is possible to observe that the elderly presented a negative effect to identify sweet and salty taste. In the sweet taste model, the sleep time promoted a positive effect in the taste identification. In the salty taste model Pittsburgh and sleep efficiency were adjust variables.

**Table 3:** Multiple regression models of sweet and salty taste with independent variables.

Model	Independent Variables	Standardized coefficients (β)	*p*	Model R^2^
1 - Sweet taste	Total Sleep Time	0.39	<0.01	0.27
	Older Adults	-0.49	<0.01	
2 - Salty taste	Older Adults	-0.25	0.04	0.13
	Pittsburgh Score^†^	0.14	0.34	
	Sleep Efficiency ^†^	-0.12	0.37	

† Adjust variables.

## DISCUSSION

In the present study, both populations evaluated presented poor sleep quality (PSQI>5), that suggest disordered sleep^19,22^. Volunteers with poor sleep quality had reduced sleep efficiency compared to those who had good sleep quality. Regarding taste sensitivity, it was verified that older adults presented difficulties in identifying sweet and salty, independent of the quality of sleep pattern.

The sleep quality comprises aspects of both objective and subjective measures, taking into account a sleep duration, sleep architecture, and subjective sleep quality. Restful sleep as one of the main indicators of good sleep quality has been suggested as a mechanism that relates to other health behaviors and well-being. Current sleep recommendations for adult are 7 to 9h/night. Other considerations included findings that older adults sleeping 6-9 hours have better cognitive functioning, lower rates of mental and physical illnesses, and enhanced quality of life compared with shorter or longer sleep durations^23^.

In addition, studies suggests sleep changes may alter chemosensory function^13,14^. According to Smith et al.^14^ taste sensitivity and dietary intake did not change after one night of sleep lasting <7h, suggesting that more than one night of sleep manipulation may be necessary to observe changes in taste sensitivity and intake.

The primary hypothesis of this study was that decreased quality of sleep would result in diminished taste sensitivity and the secondary aim was that age also impaired in diminished taste sensitivity. We observed that duration of sleep presented positive effect in the sweet taste and did not presented effect in the other tastes. Age was negative associated with sweet and salty taste sensitivity.

It was verified that older adults presented difficulties in identifying sweet and salty, independent of the quality of sleep pattern. Sensory changes are common with age. In general, this group presents decrease in gustatory and olfactory functions and it could be related with disorders of the central nervous system and the endocrine system^24,25^. According to a recent study conducted with European population, there is a great variability in the perception of the 5 basic tastes in this population^26^. The authors observed that increased age was associated with a decrease in the perception of all taste qualities, although mainly in bitter and sour tastes when population between 18 and 80 years old were evaluated. The detection thresholds for 5 basic tastes presented a significant decline with age and older men were less sensitive than young men^27^. The same authors observed that elderly needed higher concentrations to detect the compound dissolved in water^27^.

When we verified the relation of taste sensitivity with sleep time, it was demonstrated that the total sleep time presented a positive factor for the sweet taste. Smith et al.^14^ demonstrated no change in sweet or salty taste thresholds was associated with sleep deprivation, however, the preferred concentration of a sweet solution increased among habitual long sleepers after a shortened night of sleep. Moreover, in a study by Simon et al.^18^, when sleep duration was reduced in 3,5 hours, teens presented higher preference for sweet/dessert foods. So, in these studies the total sleep time is observed to be a significant predictor of taste. The study of Hogenkamp et al.^15^ carried out with young people, showed that those who slept less during the night did not have greater sensitivity to the sweet taste. According to Pardi et al.^28^, when submitted to a modest sleep loss, people may be more willing to eat food they recognize as less healthful and appear to prefer more calorically-dense foods. Hedonic responses could be a variable to be considered since sweet taste threshold could be influenced by bright light exposure^17^ and varies in adults and children^29^. How sleep architecture might contribute to differences in taste preference is currently unknown, given that the exact functions of REM and SWS are not fully understood^17^. However, despite the uncertainty of the exact mechanisms, it is clear that sleep plays a multifaceted role in biological homeostasis, particularly in the endocrine system^17^ that influence in taste sensitivity specially in elderly people.

Nevertheless, some limitations of this study need to be brought forward. This study did not find significant differences in the perception and dilution of the samples. This may be due to the number of tasters in each group and also to factors not assessed such as usual diet, sleep the day before the test and health status on the day of the test.

As expected, age cause changes in sleep quality and in taste sensitivity, especially in sweet and salty taste. Sweet taste sensitivity presented a relationship with sleep time in older adults’ population and there are few studies that correlated these variables. Finally, although no significant association between taste and nutritional status was found in this study, it is suggested that these variables should be considered in future studies in order to evaluate the effects of gustatory decrease on food intake and, consequently, on the nutritional status of individuals. Therefore, there is great importance that further studies in this area are carried out in order to improve the quality of life of the older adult population.
